# QoS-Aware Resource Allocation with Pilot-Aided Channel Estimation for Heterogeneous Wireless Networks

**DOI:** 10.3390/s22124545

**Published:** 2022-06-16

**Authors:** Andres Ortega, Velio Tralli

**Affiliations:** 1 Telecommunications Engineering, Center for Studies and Sustainable Development (CEDS), Universidad Tecnológica Ecotec, Guayaquil 092301, Ecuador; 2 Engineering Department, University of Ferrara—CNIT, I-44122 Ferrara, Italy; velio.tralli@unife.it

**Keywords:** resource allocation, heterogeneous networks, pilot-aided channel estimation, imperfect CSI, OFDMA systems

## Abstract

The deployment of heterogeneous networks (HetNets) is a way to increase the network capacity and release part of the traffic generated by users inside a cell to small-scale wireless networks for service. In this context, the main problem is managing the interference due to the coexistence of small cells and macro cells. In this paper, a QoS-aware Resource Allocation (RA) algorithm jointly working with admission control (AC) over a two-tier HetNet scenario is investigated in the presence of both the pilot-symbols for channel estimation and the channel estimation error. The RA algorithm allows two users, the macro cell user (CU) and small cell user (SU), to simultaneously share the same resource block. Moreover, system performance and fairness are improved by including adaptive power allocation to users over resource blocks. In the framework of RA with proportional rate constraints, a novel algorithm is designed by including the effects of pilot-aided channel estimation. The algorithm is able to distribute the same proportional rate to all CUs and SUs, even in the presence of channel estimation error. Relevant numerical results for the downlink of a two-tier HetNet with pilot-aided channel estimation show that the rate dispersion is driven to zero while the sum-rate is maximized, and the average user rate penalty with respect to a perfect-CSI scenario may rise to 20%.

## 1. Introduction

The data rate in 4G/5G/6G mobile communications has largely grown due to the inclusion of multimedia traffic, increasing in this way the interference among multiple users over wireless channels, thus making it more challenging to preserve performance in terms of *Quality-of-Service* (QoS).

A new network paradigm in the evolution to 5G systems, consisting of the Heterogeneous Networks (HetNets) [1], has been introduced in the last years. In HetNets, an underlay cellular system [2] with micro-femto cells and device-to-device (D2D) communications coexists with a macro cell to reduce the traffic and energy consumption. One of the main focuses in multi-tier networks is managing interference through suitable resource allocation (RA) schemes [3,4,5,6,7] in order to achieve maximum network throughput while guaranteeing the QoS required by the users. The complexity of the RA problem involved increases when new scenarios and new technologies, e.g., MIMO systems, are introduced along the 5G–6G path [8], and multiple types of resources have to be considered for global optimization [9].

In most research work, the performance of HetNets has been investigated with the assumption of a perfect knowledge of the wireless channel at the receivers and at the resource allocation unit of the network. However, in practical implementations, the channel of each wireless link is estimated through some specific techniques, e.g., pilot-aided channel estimation, that introduce some overhead in the transmission and also a channel estimation error that degrades the performance of each link in terms of the achievable rate [10,11,12,13,14,15]. Moreover, if the resource allocation algorithms do not properly take into account such performance degradation, they may fail to optimize the network throughput and to provide a fair QoS to all the users. This paper tries to address this issue by investigating QoS-aware RA algorithms that are able to operate in the presence of pilot-symbols for channel estimation and channel estimation errors.

### 1.1. Contribution of the Paper

In our paper, an RA algorithm is proposed for the downlink of a heterogeneous twotier network (macrocell-femtocells) where pilot-symbol-aided channel estimation is used, which introduces rate and power penalties and also a channel estimation error. The RA algorithm allows two users, the macrocell user and small cell user, to simultaneously share the same resource block and provides adaptive power allocation to users over resource blocks to improve network performance and fairness in the presence of interference.

The RA algorithm is derived as a suboptimal solution of a sum-rate maximization problem with proportional rate constraints and per cell power constraints. In this framework, a novel solution is designed by including the effects of pilot-aided channel estimation. It consists of two decoupled algorithms: one for subchannel allocation and one for power allocation. The second algorithm allows multiple iterations that converge to a stable solution. The RA algorithm jointly works with a suitable admission control (AC) that controls the admission of users to the network and the network load on a long-term basis by also taking into account the effects of pilot-aided channel estimation.

The results obtained from the proposed algorithms are compared with the results obtained from algorithms not aware of pilot-aided channel estimation effects and from algorithms working in the scenario with perfect CSI. This enables us to evaluate the impact of imperfect CSI on network and user performance and the impact of CSI awareness in the resource allocation process. The results also allow us to check the performance sensitivity to pilot symbol configurations—for example, the amount of power spent over pilots. The simulation results show that the proposed RA algorithms (with two iterations in the power allocation procedure) are able to distribute the same proportional rate to all macro cell and small cell users, with almost zero dispersions, even in the presence of channel estimation error.

### 1.2. Organization of the Paper

The paper is organized as follows. After the discussion of the related work in Section 2, the system model is presented in Section 3. The link model considering the effects of channel estimation error and pilot symbol overhead is illustrated in Section 4. All the RA algorithms are derived and presented in Section 5, whereas the AC strategy is summarized in Section 6. Section 7 illustrates the simulation results, and conclusions follows in Section 8. The main symbols and acronyms are listed in Table 1.

## 2. Related Work

Access methods play an important role in the design of a correct resource allocation strategy. In [16], for an OFDM system with adaptive coded modulation, an algorithm is proposed that combines subcarrier, bit, and power allocation. The authors assume perfect channel estimation and exclusive use of the subcarriers. They found that for a five-user scheme, the system achieves at least a 4 dB advantage on the average SNR with respect to traditional schemes. Dynamic Resource Allocation in Multiuser OFDM [17] makes full use of multiuser diversity due to the time-varying behavior of the wireless channel. The sum capacity of MU-OFDM is maximized when each subchannel is assigned to the user with the best channel-to-noise ratio.

Similarly, an optimal power allocation algorithm with a set of proportional fairness constraints is proposed in [18] in order to assure that each user can achieve a required data rate. In order to optimize the subchannel and power allocation, these two subproblems are solved independently, and the optimization problem is transformed into a linear problem to reduce the complexity. Therefore, the equally weighted sum capacity was defined as the objective function, and the proportional fairness was imposed as a set of nonlinear constraints. In [19], a Proportional-Fairness-Scheduling (PFS) algorithm is considered for NOMA systems to avoid the cases where the users with weak channel gains cannot be supported. The algorithm is defined for two users under two different criteria: maximize the sum-rate and maximize the minimum rate. The results have shown that NOMA/Max-Min can stabilize transmission rates and provide the smallest standard deviation of rates with respect to NOMA/Sum proposed in [20].

Resource allocation for heterogeneous networks has been considered in [1,2,3,5,6,7] referring to either a two-tier cellular network or a D2D underlaying cellular network. It is shown in [6] that spatial interference coordination can improve the spectral and energy efficiency involved in underlay HetNets. In this context, the cooperative radio resource management in cognitive radio networks [21,22] and the joint distributed cell association and power control [3] are promising techniques for 5G systems and beyond. It is emphasized in [5] that rate-optimized communications can be obtained from the solution of large-scale—often untractable—optimization problems including resource allocation and cell association. Among tractable approaches to these problems, those based on game theory are often considered. In [23], a mean-field game theory approach has been exploited to solve the interference management problem in large-scale HetNets. The problem has been decoupled into a set of smaller localized problems to be solved at the small cells with local information only. Heterogeneous ultra-dense networks have been also investigated in [24] where solutions based on game theory are employed to reduce the imbalanced cell loads and to maximize the resource utilization. Game theory can be also used in cognitive radio applications as a low-complexity approach to manage interference and to support secure spectrum sensing, as in [25].

In [26], power control for two-tier femtocell networks has been proposed using distributed utilities for the femtocells, based on rewards dependent on the signal-tointerference plus noise ratio (SINR). The SINR equilibrium is attained using distributed power updates, and the utility adaptation provides up to 30% higher SINR in femtocells, with respect to the classic Foschini–Miljanic algorithm [27]. In [28], the interference between D2D and cellular user equipments has been considered by proposing efficient Admission Control (AC) and radio resource allocation (RA) strategies, jointly working to guarantee the QoS requirements for all users under cross-tier interference. The maximization of the average weighted sum-rate under rate constraints was considered to derive allocation algorithms.

In all the previously referenced works, perfect *channel-state-information* (CSI) has been assumed. However, in practice, the RA schemes for HetNets are influenced by CSI inaccuracy. In [29], the optimization of network capacity was investigated in the presence of CSI uncertainty, demonstrating that equal power distribution among data subchannels can be an optimal choice. In [10], an energy-efficient resource allocation scheme for OFDMA two-way relay networks with imperfect CSI is proposed to improve the transmit power consumption with given QoS requirements. In addition, an optimal power allocation, relay selection and subcarrier pairing are derived through convex optimization techniques, where the related problem is solved with zero-optimal gaps, as in [11]. However, all the authors did not consider the effects of interference, which can make the problem nonconvex, and relaxation techniques are needed to solve it, as in [30,31]. When the CSI error is not considered in the RA, sharing the resource blocks (RB) may cause unpredicted interference to users.

The effects of imperfect CSI are considered in [13] for cognitive multi-radio-access HetNet. Here, the energy-efficiency optimization [32] is enhanced by properly splitting the traffic into different radio accesses to guarantee the outage probability requirements in femtocells. In [14], an optimal outage probability threshold has been derived using a probabilistic scheme, and a QoS-aware RA for D2D underlay systems has been proposed. Here, the authors considered outage-based QoS requirements and the interference to cellular users only, whereas the imperfect CSI in the interfering links is modeled as the distance-based path loss.

A two-tier HetNet with underlay D2D communications has been investigated in [12,15], and power control algorithms have been derived by taking channel estimation error into account. In [12], optimal power is derived using a primal-dual optimization method to maximize the SINR of D2D links while maintaining a minimum required QoS, whereas in [15], both centralized and distributed methods are considered to maximize coverage probability. A two-tier Hetnet with femtocells is investigated in [33], where the effects of pilot-aided channel estimation have been considered in the design of admission control, which reduces the number of admitted users in order to satisfy the QoS constraints. However, the RA issues have not been addressed in this work.

## 3. System Model

**Figure 1:**
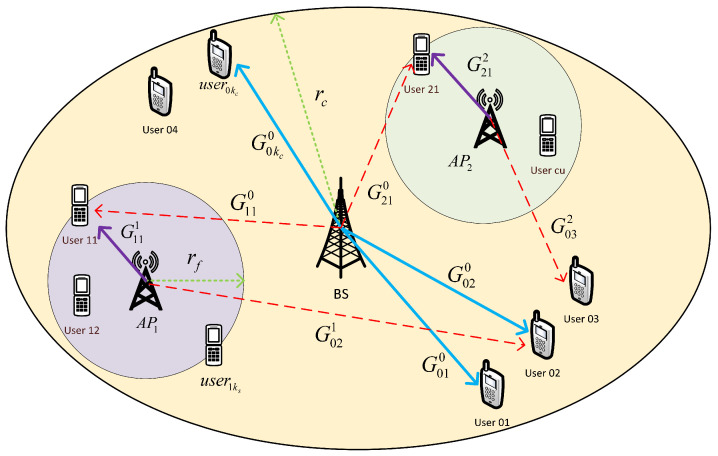
HetNet model for the downlink scenario. The lines with arrows denote the wireless links: direct link (solid), interfering link (dashed).

We consider a two-tier cellular network using OFDMA, as in Figure 1. In the network, there are $K_{c}$ cellular users (CUs), uniformly distributed in the macrocell and connected to the base station (BS); $K_{s}F$ small-cell users (SUs), each one assigned to a small-cell; and *F* small-cells. The small-cell access points (AP) are uniformly placed in the macrocell region limited by the circles of radio $r_{i}$ and $r_{c}$, where $r_{c}$ is the macrocell radius and $r_{i}$ is a minimum distance of CUs to BS. The SUs are uniformly distributed inside the small-cells, at $K_{s}$ per cell. The small-cell radius is $r_{f}$. We identify the users with a pair of indexes (c,u): primary cellular users with c=0 and $u=1,\ldots ,K_{c}$; small-cell users with c>0 and $u=1,\ldots ,K_{s}$). Each user is assumed to be preassigned to cell c∈{0,1,…,F}, where c=0 is the macrocell.

The available OFDMA bandwidth is divided into S orthogonal subchannels with bandwidth ΔB, where the elementary resource unit is the resource block (RB), composed of one subchannel and one time slot. Each subchannel can include several subcarriers: e.g., in 4G numerology, one subchannel includes 12 OFDM subcarriers with ΔB=180 KHz. All the available RBs are shared among the coexisting CUs and SUs communications. We assume that the network is fully loaded and the SUs can only be served in sharing (not orthogonal) mode with CUs. Thus, an RB can be allocated to either a CU in orthogonal mode or to a coupled CU and SU in sharing mode. Sharing mode communications are affected by cross-interference, as in the case illustrated in Figure 1 for the downlink scenario.

The network supports a QoS-aware centralized resource management that controls resource assignment to all users in order to limit the effects of cross-interference and optimize the revenue for the service provider while guaranteeing a predefined level of QoS to users. The QoS requirement is defined in terms of minimum average bit-rate, denoted with $q_{cu}$, $u=1,\ldots ,K_{c}\left(K_{s}\right)$, (c=0,…,F). The related revenue for the service provider is denoted with $w_{cu}$ for $u=1,\ldots ,K_{c}\left(K_{s}\right)$, (c=0,…,F).

**Figure 2:**
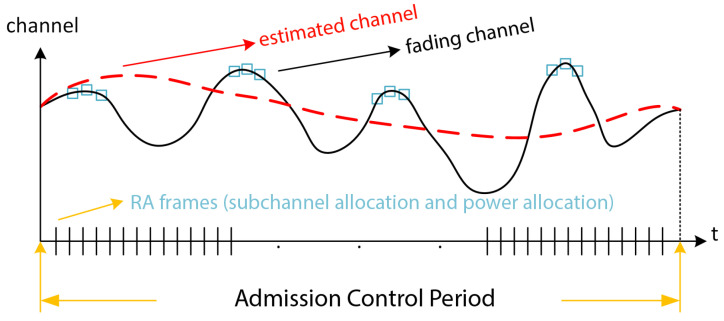
Time scale of admission control and resource allocation with respect to channel variations.

We use a dynamic radio resource management due to the time-varying nature of the wireless channel, as sketched in Figure 2. This is is based on a radio resource allocation (RA) algorithm that efficiently allocates RBs and controls the power, frame by frame, and an admission control (AC) algorithm that determines which users can be served by the network in a long-term period under QoS requirements. The RA algorithm sees short-term time-varying fading conditions, even in the frequency domain. The AC algorithm sees average channel conditions in its operating period. It admits the users that have the best channel conditions in the long-term.

If the system is properly loaded by the AC algorithm, the RA algorithm works to guarantee the QoS requirement. More specifically, as better explained in Section 4, the RA algorithm tries to maximize frame-by-frame the bit-rate of each user while preserving proportionality to rate requirements. In this case, the service provider revenues depend on the outcome of the AC algorithm. If we use the binary variable $z_{cu}$ to indicate whether (1) or not (0) the CUs and the SUs are admitted in the system, respectively, the network utility revenues become

(1) $$U\left(z\right)=\sum_{c=1}^{F}\sum_{u=1}^{K_{s}}z_{cu}w_{cu}+\sum_{u=1}^{K_{c}}z_{0u}w_{0u}$$

where $z=\{z_{cu}:c=0,\ldots ,F,u=1,\ldots ,K_{c}\left(K_{s}\right)\}$ is the outcome of the AC algorithm.

### Channel Model

**Figure 3:**
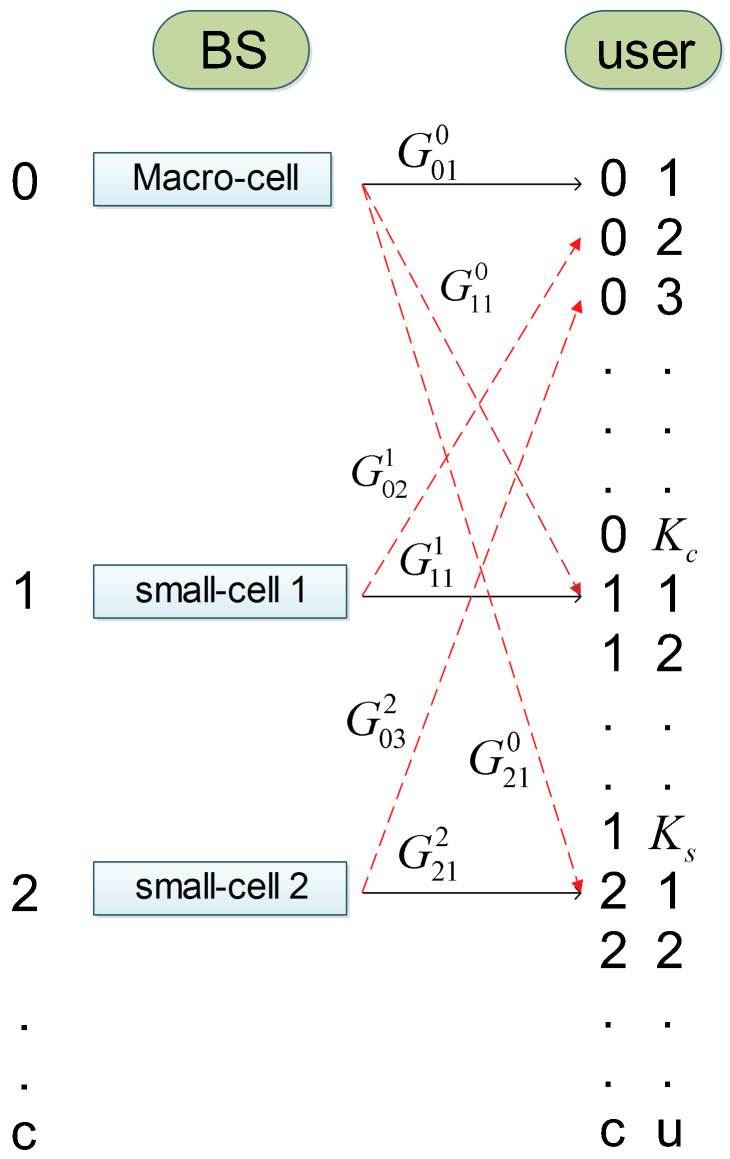
Notations for the channel gains of the wireless links between base stations and users.

The wireless channel between each transmitter–receiver couple is modeled through a distance-dependent path loss and a random small-scale fading, which is assumed to be constant along the set of consecutive slots of a frame. By taking the downlink as a reference case, the power received by the user *u* of cell *c*, from the base station or access point *b*, on the OFDM subchannel *s* is given by $P_{R,cus}^{b}=P_{us}^{c}\cdot G_{cu}^{b}\cdot h_{cu,s}^{b}$, where $G_{cu}^{b}$ represents the average channel gain and $h_{cu,s}^{b}$ represents the random fading gain on the subchannel *s*. The notation scheme is illustrated in Figure 3. The average channel gain is the multiplicative inverse of the path-loss, which is modeled [15] as follows:

(2) $$PL\left(\mathrm{dB}\right)=\{\begin{array}{cc} 128.1+37.6\log_{10}d\left[\mathrm{km}\right] & \;\;\mathrm{BS}\to \mathrm{CU}\\ 128.1+37.6\log_{10}d\left[\mathrm{km}\right]+15 & \begin{array}{c} \;\;\mathrm{BS}\to \mathrm{SU}\\ \;\;\mathrm{AP}\to \mathrm{CU} \end{array}\\ 98.46+20\log_{10}d\left[\mathrm{km}\right]+0.7d\left[m\right] & \;\;\mathrm{AP}\to \mathrm{SU} \end{array}$$

Small-scale fading is due to multipath propagation in the wireless medium and takes different correlated values across the subchannels in the frequency domain. In this regard, we consider a multipath channel model with L+1 independent paths with zero-mean complex-Gaussian path gains with variance $p_{l}$, with l=0,…,L, and $\sum_{l}p_{l}=1$. The set of values $p_{l}$ is also referred to as the Power Delay Profile (PDP) of the channel. The low-pass transfer function of the channel, between base station *b* and user (c,u), sampled at the subchannel frequencies sΔB, with s=0,…,S−1, is given by

(3) $$H_{cu,s}^{b}=\sum_{l=0}^{L}a_{cu,l}^{b}\cdot e^{j\theta_{cu,l}^{b}}\cdot e^{-j2\pi s\Delta B\tau_{cu,l}^{b}}$$

where $a_{cu,l}^{b}$ and $\theta_{cu,l}^{b}$ represent the random amplitude and phase, respectively, of the tap *l* with delay $\tau_{cu,l}^{b}$, and $p_{l}=E\left[{\left(a_{cu,l}^{b}\right)}^{2}\right]$. The random fading gain is therefore given by $h_{cu,s}^{b}={\left|H_{cu,s}^{b}\right|}^{2}$.

## 4. Link Model

We first introduce the model of SINR as in [28], for the case when the CSI is perfectly known at the receiver. After this, we extend this model to include the effects of the redundancy introduced by pilot symbol-assisted channel estimation and the effects of channel estimation error.

When the CSI is perfectly known, the SINR for the user *u* of cell *c*, when it shares the channel with user *v* of cell *b* on subchannel *s*, is given by

(4) $$\gamma_{cus}^{\left(bv\right)}=\frac{P_{us}^{c}G_{cu}^{c}h_{cu,s}^{c}}{\sigma^{2}+P_{vs}^{b}G_{cu}^{b}h_{cu,s}^{b}},\begin{array}{c} b\neq c \end{array}$$

We assume that at most one SU can share the subchannel *s* with a CU. If the user *u* of cell *c* does not share the subchannel, i.e., it operates in orthogonal mode, the SINR misses the interference term leading to $\gamma_{cu,s}^{\prime }=P_{us}^{c}G_{cu}^{c}h_{cu,s}^{c}/\sigma^{2}$. We use symbol $\gamma^{\prime }$ to denote this specific case.

When pilot-symbol-aided CSI estimation is implemented to allow OFDM coherent detection and synchronization, a part of the transmitted symbols in each RB is used as pilots, thus reducing the number of symbols and the amount of power available for the transmission of useful data. Moreover, since channel estimation is not perfect, a residual estimation error acts as additional noise that degrades the link performance.

We assume that a minimum mean square error (MMSE) estimator is used at each receiver to estimate the impulse response of the frequency-selective channel, which is optimal with respect to link capacity. It is shown in [34] that the mean square error, i.e., the variance of the estimation error, for the generic user *u* of cell *c* in case of equipowered and equispaced pilots, is given by

(5) $$\sigma_{cu}^{2}=\sum_{l=1}^{L}{\left[\frac{1}{p_{l}}+\frac{P_{Pcu}}{\sigma_{n}^{2}}\right]}^{-1}$$

where $P_{Pcu}$ is the total average power received over pilot symbols, $\sigma_{n}^{2}$ is the noise power per subcarrier, and $\left\{p_{0},\ldots ,p_{L}\right\}$ is the PDP of the channel with *L* paths. In a general case, we have $\sigma^{2}=N_{sc}\sigma_{n}^{2}$, where $N_{sc}$ is the number of subcarriers per RB, e.g., $N_{sc}=12$ in 4G numerology.

It is also shown in [34] that the instantaneous (short-term) signal-to-noise ratio in a given subcarrier (or RB in our case) at the receiver is given by

(6) $$\rho =\frac{P_{D}^{\left(\mathrm{RB}\right)}\left(1-\sigma_{e}^{2}\right)g}{\sigma^{2}+P_{D}^{\left(\mathrm{RB}\right)}\sigma_{e}^{2}}$$

where $P_{D}^{\left(\mathrm{RB}\right)}$ is the average power received over the data symbols of the RB, *g* is the instantaneous fading variable, with unit mean, in the given subcarrier (RB in our case), and $\sigma_{e}^{2}$ is the variance of the estimation error.

We apply these results to reformulate the SINR in (4) for a system with pilot-symbol assisted CSI estimation. By including the short-term and the long-term average components of (6) and by including the interference, the SNIR for user *u* of cell *c*, when it shares the channel with user *v* of cell *b* on the subchannel *s*, becomes as follows:

(7) $$\gamma_{cus}^{\left(bv\right)}=\frac{P_{us}^{c}G_{cu}^{c}h_{cu,s}^{c}\left(1-\sigma_{cu}^{2}\right)\eta_{p}}{\sigma^{2}+P_{us}^{c}G_{cu}^{c}\sigma_{cu}^{2}\eta_{p}+P_{vs}^{b}G_{cu}^{b}h_{cu,s}^{b}\eta_{p}}$$

where $\eta_{p}$ is the power efficiency, i.e., the ratio between the power used over RB data symbols and the total power $P_{us}^{c}$. In orthogonal mode, the SINR becomes

(8) $$\gamma_{cus}^{\prime }=P_{us}^{c}G_{cu}^{c}h_{cu,s}^{c}\left(1-\sigma_{cu}^{2}\right)\eta_{p}/\left(\sigma^{2}+P_{us}^{c}G_{cu}^{c}\sigma_{cu}^{2}\eta_{p}\right)$$

The average rate achievable by the user *u* of cell *c* over one slot is therefore given by

$$R_{cu}=\sum_{s}a_{cus}A_{3}\eta_{b}\Delta B[{\bar{a}}_{cs}\log_{2}(1+\gamma_{cus}^{\prime }/A_{1})+\sum_{b\neq c,v}a_{bvs}\log_{2}(1+\gamma_{cus}^{\left(bv\right)}/A_{1})]$$

where the parameters $A_{1}$ and $A_{3}$ are the SNR-gap and the rate adjustment, respectively, depending on the adaptive modulation and coding (AMC) used at the physical layer, $\eta_{b}$ is the bandwidth efficiency of pilot-aided channel estimation, and $a_{cus}$ is the allocation variable of user *u* of cell *c* for the subchannel *s*. The allocation variable $a_{cus}$ is 1 when the subcarrier is allocated to the user; it is 0 otherwise. Similarly, the orthogonal-mode allocation variable ${\bar{a}}_{cs}=1-\sum_{b\neq c,v}a_{bvs}$ is 1 when the subchannel *s* is not allocated to any user of the cells b≠c; it is 0 otherwise, noting that at most one of the users of the cells b≠c can share the subchannel.

The bandwidth efficiency is given by the ratio between the number of data symbols and the total number of symbols in one RB, which can also be written as

(9) $$\eta_{b}=1-\mu =1-N_{P}/N$$

where $N_{P}$ is the number of pilots in one RB and *N* the total number of symbols in one RB.

To introduce flexibility in the use of pilots, we consider the possibility to allocate different amounts of power to pilot and data symbols. If we used the same power level for all symbols, i.e., a power $\bar{P}$, the power allocated to pilots would simply be $\mu \bar{P}$. To differentiate the power level for pilots, we allocate to them a power equal to $\alpha \mu \bar{P}$, where α is a power allocation parameter that can be different from 1. In this way, the power efficiency becomes

(10) $$\eta_{p}=1-\alpha \mu$$

According to these definitions, the total power received over pilot symbols by the user *u* of cell *c* in the downlink (note that in the downlink, each receiver is able to process pilots spread over the entire bandwidth SΔB) becomes

(11) $$P_{Pcu}=\alpha \cdot \mu \cdot S\cdot {\bar{P}}_{c}\cdot G_{cu}^{c}$$

where $S{\bar{P}}_{c}$ is the total power available at the BS or AP *c*, and ${\bar{P}}_{c}$ is the power per RB.

Finally, with $\eta_{cus}=a_{cus}A_{3}\eta_{b}\Delta B$, the rate becomes

$$R_{cu}=\sum_{s}\eta_{cus}[{\bar{a}}_{cs}\log_{2}\left(1+\gamma_{cus}^{\prime }/A_{1}\right)+\sum_{b\neq c,v}a_{bvs}\log_{2}\left(1+\gamma_{cus}^{\left(bv\right)}/A_{1}\right)]$$

## 5. Resource Allocation

The aim of the RA algorithms is to efficiently assign to the admitted CUs and SUs, in each frame, the available RBs and the available power. We define for each cell *c* the set of admitted users as ${Α}_{c}=\left\{u:z_{cu}=1\right\}$. The RA frame includes *S* RBs ×*T* slots. For the sake of simplicity, in the RA problem formulation, we extend the subchannel index range to cover the entire frame (in this case, the actual user rate becomes $R_{cu}/T$), i.e., s=1,…,ST, and assume that random fading gains remain constant in the adjacent slots of the frame. The RA algorithm tries to maximize frame-by-frame the bit-rate of each user while preserving proportionality to rate requirements.

The optimization problem which is considered for the RA can described as follows:

(12a) $$\max_{A,P}\;\sum_{c}\sum_{u}R_{cu}$$

(12b) $$s.t.\;:\;a_{cus}\in \left\{0,1\right\}$$

(12c) $$\sum_{u}a_{0us}\leq 1\;\forall s$$

(12d) $$\sum_{c>0}\sum_{u}a_{cus}\leq 1\;\forall s$$

(12e) $$P_{us}^{c}\geq 0$$

(12f) $$\sum_{s}\sum_{u}a_{cus}P_{us}^{c}\leq P_{T}^{c},\;\forall c$$

(12g) $$\frac{R_{cu}}{\phi_{cu}}=\frac{R_{c1}}{\phi_{c1}}\;\forall u>1,\forall c$$

(12h) $$\frac{R_{c1}}{\phi_{c1}}\leq \frac{R_{01}}{\phi_{01}}\;\forall c>0$$

where $P=\{P_{us}^{c},c=0,\ldots ,F;u\in {Α}_{c};s=1,\ldots ,ST\}$, $A=\{a_{cus},c=0,\ldots ,F;u\in {Α}_{c};s=1,\ldots ,ST\}$ and $\phi_{cu}=q_{cu}$ is the proportionality constant linked to the QoS requirements defined in Section 2 in terms of the minimum average bit rate. We assume, for the sake of simplicity, that we renumber the user indexes $u\in {Α}_{c}$ to have them in the range $\left[1,|{Α}_{c}|\right]$.

In problem (12), the constraints b, c and d are related to the allocation variables: each subchannel can be allocated to no more than one CU and one SU. The constraints e and f are related to the allocated powers: their sum cannot exceed the power budget $P_{T}^{c}$ (per frame) at each BS or AP. The constraints g and h ensure rate proportionality among CUs and SUs: the proportionality is strict within each cell, but the proportional rate allocated in each small cell may be less than the proportional rate in the macrocell, when the power budget of each small cell does not support larger rates.

The problem in (12) is a mixed integer programming problem with non-convex object and constraints. Due to the problem complexity, we seek for a suboptimal solution by decomposing the problem into two decoupled sub-problems:

- A subchannel allocation problem defined by assuming an equal power distribution among subcarriers;
- A power allocation problem defined by assuming the subchannels already allocated according to the solution of the previous problem.

We follow the suboptimal low-complexity approach proposed in [18] for a single-cell network with perfect CSI knowledge and extend it to our heterogeneous network with imperfect pilot-symbol assisted CSI estimation. We should remind the reader that the main scope of the paper is to evaluate the impact of imperfect CSI on the resource allocation process. Nevertheless, we also provide, as a result of this extension, a novel suboptimal low-complexity algorithm for RA in a two-tier cellular system.

### 5.1. Subchannel Allocation Algorithm

We first discuss the suboptimal subchannel allocation algorithm, which is described by Algorithm 1. It is based on two main successive steps that follow the initialization step. In the first step, the subchannel allocation to all the CUs is performed. All the channels are assigned to the CUs trying to maximize the sum-rate while preserving proportional fairness. This part of the algorithm is similar to the algorithm proposed in [18], Section IV. In the second step, which is novel with respect to [18], the subchannel allocation to all the SUs in sharing mode is done. In this part of the algorithm, the sum-rate is maximized by having in mind that when a channel is assigned to an SU in sharing mode with a CU, the rate of the CU decreases while the rate of the SU increases.

The specific details of Algorithm 1 are as follows. In step 2.a, one subchannel is allocated to each CU by selecting the subchannel with the best channel gain in the set of still unassigned channels. In step 2.b, the remaining unassigned channels are allocated according to the rule that, in each turn, the user with the smallest proportional rate gets the priority to choose their best subchannel. In step 3.a, one subchannel is allocated to each SU in sharing mode by selecting the subchannel with the best rate–fairness tradeoff in the set of still unassigned channels. The rate–fairness tradeoff due to the allocation of subchannel *s* to SU (c,u) is evaluated with the rate metric:

(13) $$m_{cu,s}=\min\left(R_{cu}^{\prime }/\phi_{cu},R_{0k}^{\prime }/\phi_{0k}\right)$$

where k=σ(s) is the index of the CU sharing the subchannel *s*, and $R_{cu}^{\prime }$, $R_{0k}^{\prime }$ are rates updated with the possible allocation of subchannel *s* in sharing mode. Finally, in step 3.b, the remaining unassigned (in sharing mode) channels are allocated according to the rule that, in each turn, the user with the smallest proportional rate gets the priority to choose their best subchannel in terms of best rate–fairness tradeoff. The subchannel is assigned only if the rate metric is equal to or larger than the proportional rate of the SU before subchannel allocation.
**Algorithm 1** Subchannel allocation algorithm.Step 1Initialize $R_{cu}=0$, $a_{cus}=0$, ∀c,u,s; Initialize set of channels for CUs and SUs $\Omega_{CU}=\Omega_{SU}=\left\{1,\ldots ,ST\right\}$;Step 2**Allocate subchannels to all CUs**;(a)**for**u=1 to $K_{C}$find *n* satisfying $h_{0u,n}^{0}\geq h_{0u,s}^{0}$, $\forall s\in \Omega_{CU}$;set $a_{0un}=1$, $\Omega_{CU}=\Omega_{CU}-\left\{n\right\}$;update $R_{0u}$ according to (9);    **endfor**(b)**while**$\Omega_{CU}\neq \emptyset$find *k* satisfying $\frac{R_{0k}}{\phi_{0k}}\leq \frac{R_{0u}}{\phi_{0u}},\forall u$;find *n* satisfying $h_{0k,n}^{0}\geq h_{0k,s}^{0}$, $\forall s\in \Omega_{CU}$;set $a_{0kn}=1$, $\Omega_{CU}=\Omega_{CU}-\left\{n\right\}$;update $R_{0k}$ according to (9);    **endwhile**Step 3**Allocate subchannels to all SUs [in sharing mode with CUs]**;(a)**for**c=1 to *F*, u=1 to $K_{S}$find *n* satisfying $m_{cu,n}\geq m_{cu,s}$, $\forall s\in \Omega_{SU}$;set $a_{cun}=1$, $\Omega_{SU}=\Omega_{SU}-\left\{n\right\}$, k=σ(n);update $R_{cu}$, $R_{0k}$ according to (9);   **endfor**(b)initialize set C={(c,u),∀c≠0,∀u}    **while** C≠∅ and $\Omega_{SU}\neq \emptyset$  find (b,v) satisfying $\frac{R_{bv}}{\phi_{bv}}\leq \frac{R_{cu}}{\phi_{cu}},\forall \left(c,u\right)\in C$;  find *n* satisfying $m_{bv,n}\geq m_{bv,s}$, $\forall s\in \Omega_{SU}$;     **if** $m_{bv,n}\geq \frac{R_{bv}}{\phi_{bv}}$ **then**  set $a_{bvn}=1$, $\Omega_{SU}=\Omega_{SU}-\left\{n\right\}$;  set k=σ(n);  update $R_{bv},R_{0k}$ according to (9);     **else**  set C=C−{(b,v)};     **endif**     **endwhile**

### 5.2. Power Allocation Algorithm

The power allocation algorithm is derived by assuming that subchannel allocation, identified by the set **A** $=\{a_{cus}^{*}\}$, is the result of Algorithm 1. By considering the general problem in (12), the power allocation problem can be formulated as

(14a) $$\begin{array}{cc} \max_{P}\; & \sum_{c}\sum_{u}R_{cu} \end{array}$$

(14b) $$\begin{array}{cc} \;\;\;\;\;s.t.:\; & P_{us}^{c}\geq 0 \end{array}$$

(14c) $$\begin{array}{cc} \;\;\;\;\;\; & \sum_{s}\sum_{u}a_{cus}^{*}P_{us}^{c}\leq P_{T}^{c},\;\forall c \end{array}$$

(14d) $$\begin{array}{cc} \;\;\;\;\;\; & \frac{R_{cu}}{\phi_{cu}}=\frac{R_{c1}}{\phi_{c1}}\;\forall u>1,\forall c \end{array}$$

(14e) $$\begin{array}{cc} \;\;\;\;\;\; & \frac{R_{c1}}{\phi_{c1}}\leq \frac{R_{01}}{\phi_{01}}\;\forall c>0 \end{array}$$

Due to the presence of the interference term inside the expressions of the rates $R_{cu}$, this optimization problem is non-convex. A simple suboptimal solution can be derived by assuming all the interference terms to be fixed to a constant value. This can be effectively done by replacing the interfering powers $P_{vs}^{b}$ in the SINR of Equation (7) with the constant value $P_{T}^{b}/\sum_{s}\sum_{v}a_{bvs}^{*}$ = ${\bar{P}}_{b}$. With this assumption, the problem become convex and can be solved with standard methods.

To simplify the derivation, we modify the notation for the expression of the rate $R_{cu}$ of user *u* in cell *c*, evaluated through (7) and (12), as follows:

(15) $$R_{cu}=\sum_{s}\eta_{cus}^{*}\log_{2}\left(1+\Gamma_{cus}/A_{1}\right)$$

(16) $$\Gamma_{cus}=\frac{P_{us}^{c}G_{cu}^{c}h_{cus}^{v}\left(1-\sigma_{cu}^{2}\right)\eta_{p}}{\sigma^{2}+P_{us}^{c}G_{cu}^{c}\sigma_{cu}^{2}\eta_{p}+\sum_{b\neq c}\sum_{v}a_{bvs}^{*}P_{vs}^{b}G_{cu}^{b}h_{cus}^{b}\eta_{p}}$$

The notation of (16) can be further simplified as

(17) $$\Gamma_{cus}=\frac{P_{us}^{c}X_{cus}}{P_{us}^{c}Y_{cu}+Z_{cus}}$$

where

$$\begin{array}{c} X_{cus}=G_{cu}^{c}h_{cus}^{c}\left(1-\sigma_{cu}^{2}\right)\eta_{p}\\ Y_{cu}=G_{cu}^{c}\sigma_{cu}^{2}\eta_{p}\\ Z_{cus}=\sigma^{2}+\sum_{b\neq c}\sum_{v}a_{bvs}^{*}{\bar{P}}_{b}G_{cu}^{b}h_{cus}^{b}\eta_{p} \end{array}$$

including the replacement of interfering powers $P_{vs}^{b}$ with the constant value ${\bar{P}}_{b}$.

As shown in Appendix A, a first step to solving the convex problem with constant interference can be the evaluation of the Lagrangian of the problem, as a function of the set of powers P. It is found that the set of powers that maximizes the Lagrangian satisfies the following property:

**Lemma** **1.** For any user u of cell c, given the set of its allocated subchannels, i.e., $S_{cu}=\left\{s:a_{cus}^{*}=1\right\}$, the following relationship holds for any pair of allocated subchannels $s,r\in S_{cu}$:

(18) $$\begin{array}{c} \frac{X_{cus}Z_{cus}}{{\left(P_{us}^{c}\right)}^{2}Y_{cu}\left(X_{cus}+x\right)+P_{us}^{c}Z_{cus}\left(X_{cus}+y\right)+A_{1}{Z_{cus}}^{2}}=\\ \frac{X_{cur}Z_{cur}}{{\left(P_{ur}^{c}\right)}^{2}Y_{cu}\left(X_{cur}+x\right)+P_{ur}^{c}Z_{cur}\left(X_{cur}+y\right)+A_{1}{Z_{cur}}^{2}} \end{array}$$

*where*$x=A_{1}Y_{cu}$*and*$y=2A_{1}Y_{cu}$*. This relationship holds only if*$P_{us}^{c}>0$*and*$P_{ur}^{c}>0$.

Note that when the channel estimation error is not considered in the allocation algorithm, i.e., $\sigma_{cu}^{2}=0$, $Y_{cu}$ becomes 0, leading to the simplified relationship:

(19) $$\frac{X_{cus}}{P_{us}^{c}X_{cus}+A_{1}Z_{cus}}=\frac{X_{cur}}{P_{ur}^{c}X_{cur}+A_{1}Z_{cur}}$$

The relationship in (18) allows, for each user, the power to be derived to spend on each subcarrier as function of the power assigned to a reference subchannel of the user, which can be, for example, the allocated subchannel with the smallest index or the subchannel with the best SINR. If we denote with $r_{cu}$ the index of the reference subchannel for the user *u* of cell *c* and with $p_{cu}$ the power assigned to this subchannel, we can obtain $P_{us}^{c}$ as the solution of Equation (18) with $r=r_{cu}$ and $P_{ur}^{c}=p_{cu}$. This solution is derived in the Appendix A and is given by

(20) $$P_{us}^{c}=F_{us}^{c}\left(p_{cu},r_{cu}\right)$$

where function $F_{us}^{c}\left(p,r\right)$ is defined in (A6) of the Appendix A.

Now, the solution of the power allocation problem with constant interference can be obtained as the solution of the set of constraints in Equations (14c)–(14e) with the unknown $p_{cu}$, which becomes

(21a) $$\begin{array}{cc} \;\;\;\;\;\; & \sum_{u}Q_{0u}=P_{T}^{0} \end{array}$$

(21b) $$\sum_{u}Q_{cu}\leq P_{T}^{c}\;\forall c>0$$

(21c) $$\frac{R_{cu}}{\phi_{cu}}=\frac{R_{c1}}{\phi_{c1}}\;\forall u>1,\forall c$$

(21d) $$\frac{R_{c1}}{\phi_{c1}}\leq \frac{R_{01}}{\phi_{01}}\;\forall c>0$$

where $Q_{cu}=\sum_{s}a_{cus}^{*}F_{us}^{c}\left(p_{cu},r_{cu}\right)$ and

(22) $$R_{cu}=\sum_{s}\eta_{cus}^{*}\log_{2}\left(1+\frac{1}{A_{1}}\frac{F_{us}^{c}\left(p_{cu},r_{cu}\right)X_{cus}}{F_{us}^{c}\left(p_{cu},r_{cu}\right)Y_{cu}+Z{}_{cus}}\right)$$

It should be remarked that the power constraint for the CUs (c=0) is active with equality to guarantee the maximum rate to both CUs and SUs. However, the power constraints (21b) and the rate constraints (21d) for the SUs (c>0) are competing with each other, and only one of them for each small cell *c* will be active: depending on the power budget $P_{T}^{c}$, the capacity of the small cell *c* will be limited by the available power or by the rate constraint.

The power allocation algorithm is described by Algorithm 2. After the initialization of parameters, functions and indexes of reference subcarriers, in Step 3, the powers of the CUs (users of cell 0) are derived as the solution of the set of equations obtained from (21c), with c=0, and from (21a). In Step 4, for each small cell *c*, a first attempt is made to derive the power of the users by solving the equations obtained from (21c) and (21d), for c>0. In Step 5, the constraint (21b) is evaluated and checked for each cell c>0. If the power constraint is not satisfied, the powers of the SUs of cell *c* are derived as the solution of the set of equations obtained from (21c), with c>0, and from (21b). All the solutions of the non linear equations and of the set of equations are found with numerical root-finding methods.

We finally remind the reader that the power allocation algorithm provides suboptimal solutions for the power allocation problem in (14), obtained by fixing the interfering power in the SINR terms. These solutions can be improved with multiple iterations of Algorithm 2, where at each iteration, the interfering powers are updated by using the allocated powers derived at the previous iteration. An additional update can even be performed between Steps 3 and 4. It is seen that very few iterations are needed to converge to the optimal solution.
**Algorithm 2** Power allocation algorithm.Step 1Initialize $X_{cus}$, $Y_{cu}$, $Z_{cus}$, ∀c,u,s; define function in (A6);Step 2For each user (c,u), define $r_{cu}$ as the subchannel with the best SINR;Step 3Find the solutions $\left\{p_{0u},u=1,\ldots ,K_{c}\right\}$ of the set of equations
$$\left\{\begin{array}{c} \sum_{s}\frac{\eta_{0us}^{*}}{\phi_{0u}}\log_{2}\left(1+\frac{1}{A_{1}}\frac{F_{us}^{0}\left(p_{0u},r_{0u}\right)X_{0us}}{F_{us}^{0}\left(p_{0u},r_{0u}\right)Y_{0u}+Z_{0us}}\right)=\sum_{s}\frac{\eta_{01s}^{*}}{\phi_{01}}\log_{2}\left(1+\frac{1}{A_{1}}\frac{F_{1s}^{0}\left(p_{01},r_{01}\right)X_{01s}}{F_{1s}^{0}\left(p_{01},r_{01}\right)Y_{01}+Z_{01s}}\right),\;\forall u>1\\ \sum_{u}\sum_{s}a_{0us}^{*}F_{us}^{0}\left(p_{0u},r_{0u}\right)=P_{T}^{0} \end{array}\right.$$Evaluate the powers $P_{us}^{0}$, ∀u,s:
$$P_{us}^{0}=\left\{\begin{array}{cc} 0 & \mathrm{if}\;a_{0us}^{*}=0\\ F_{us}^{0}\left(p_{0u},r_{0u}\right) & \mathrm{if}\;a_{0us}^{*}=1 \end{array}\right.$$Evaluate the rate $R_{01}$Step 4**for**  c=1 to *F*  **for** u=1 to $K_{s}$    Find the solution $p_{cu}$ of the equation
$$\sum_{s}\frac{\eta_{cus}}{\phi_{cu}}\log_{2}\left(1+\frac{1}{A_{1}}\frac{F_{us}^{c}\left(p_{cu},r_{cu}\right)X_{cus}}{F_{us}^{c}\left(p_{cu},r_{cu}\right)Y_{cus}+Z_{cus}}\right)=R_{01}$$    Evaluate the powers $P_{us}^{c}$, ∀s:
$$P_{us}^{0}=\left\{\begin{array}{cc} 0 & \mathrm{if}\;a_{cus}^{*}=0\\ F_{us}^{c}\left(p_{cu},r_{cu}\right) & \mathrm{if}\;a_{cus}^{*}=1 \end{array}\right.$$  **endfor****endfor**Step 5**for**c=1 to *F*    Evaluate: $P=\sum_{s}\sum_{u}a_{cus}^{*}\left(P_{us}^{c}\right)$  **if** $P>P_{T}^{c}$    Find the solutions $\left\{p_{cu},u=1,\ldots ,K_{s}\right\}$ of the set of equations
$$\;\;\left\{\begin{array}{c} \sum_{s}\frac{\eta_{cus}^{*}}{\phi_{cu}}\log_{2}\left(1+\frac{1}{A_{1}}\frac{F_{us}^{c}\left(p_{cu},r_{cu}\right)X_{cus}}{F_{us}^{c}\left(p_{cu},r_{cu}\right)Y_{cu}+Z_{cus}}\right)=\sum_{s}\frac{\eta_{c1s}^{*}}{\phi_{c1}}\log_{2}\left(1+\frac{1}{A_{1}}\frac{F_{1s}^{c}\left(p_{c1},r_{c1}\right)X_{c1s}}{F_{1s}^{c}\left(p_{c1},r_{c1}\right)Y_{c1}+Z_{c1s}}\right),\;\forall u>1\\ \sum_{u}\sum_{s}a_{cus}^{*}F_{us}^{c}\left(p_{cu},r_{cu}\right)=P_{T}^{c} \end{array}\right.$$    Evaluate the powers $P_{us}^{c}$, ∀u,s:
$$P_{us}^{c}=\left\{\begin{array}{cc} 0 & \mathrm{if}\;a_{cus}^{*}=0\\ F_{us}^{c}\left(p_{cu},r_{cu}\right) & \mathrm{if}\;a_{cus}^{*}=1 \end{array}\right.$$  **endif****endfor**

## 6. Admission Control

The aim of the AC is to select a set of CUs and SUs that can be supported by the heterogeneous cellular network with a guaranteed QoS, i.e., a long-term average rate, and that maximize the total revenue of the provider. In this work, we consider a reformulated version the AC algorithm originally proposed in [28] for a cellular system with underlying device-to-device communications. This reformulated version takes into account the HetNet scenario and the effects of pilot-aided channel estimation. In this section, we illustrate the optimization problem from which the algorithm is derived. The details of the algorithm, which is a low-complexity greedy algorithm based on clustering and iterative linear programming achieving a solution near to the optimum, can be found in [28].

The basic assumption behind AC is the validity of a simple model for the evaluation of the average bit-rate achievable over one RB when the RB is not shared with any other user, i.e., it is used in orthogonal mode, or when the RB is shared with one of the users of the other cellular tier. This model is what we call a "long-term rate model". Let us denote with $r_{cu}^{\left(bv\right)}$ the long-term average rate of user *u* of cell *c* when it shares the RB with user *v* of cell *b*. When user *u* of cell *c* operates in orthogonal mode, its average rate is denoted with $r_{cu}^{\prime }$.

In an OFDMA multi-user scenario, the long-term achievable data rate is dependent on the statistical distribution and on the correlation of the short-term fading in all useful and interfering links. Since its evaluation is a hard task, the long-term data-rate is usually estimated (e.g., in [35]) by considering the average channel conditions of direct and interfering links and by also taking into account the multi-user diversity gain captured by the underlaying RA. This estimation is sufficiently reliable for the AC when the underlying RA algorithm assigns the resources by maximizing a weighted average sum rate with a QoS constraint for each single CU or SU.

Based on that, the long-term average rate $r_{cu}^{\left(bv\right)}$ is estimated as

(23) $$r_{cu}^{\left(bv\right)}=A_{3}\eta_{b}\Delta B\log_{2}\left(1+A_{2}\Upsilon_{cu}^{b}\right)$$

where the approximated SINR is given by

(24) $$\Upsilon_{cu}^{b}=\frac{{\bar{P}}_{c}G_{cu}^{c}\left(1-\sigma_{cu}^{2}\right)\eta_{p}}{\sigma^{2}+{\bar{P}}_{c}G_{cu}^{c}\sigma_{cu}^{2}\eta_{p}+{\bar{P}}_{b}G_{cu}^{b}\eta_{p}}$$

which does not depend on the channel conditions of interfering user *v* in the downlink. In the two equations above, ${\bar{P}}_{c}$ is the power per RB available at the BS or AP *c*, and the parameter $A_{2}$ accounts for the multiuser diversity gain captured by the RA algorithm and the SNR-gap of the AMC used at the physical layer. According to [28], it is defined as $A_{2}=\varsigma \ln\left(K_{c}\right)/A_{1}$ and $A_{2}=\varsigma \ln\left(FK_{s}\right)/A_{1}$ for the rate evaluation of CUs and SUs, respectively, where ς is a tuning parameter. If the user *u* of cell *c* has exclusive use of the RB in orthogonal mode, the average rate is estimated as

(25) $$r_{cu}^{\prime }=A_{3}\eta_{b}\Delta B\log_{2}\left(1+A_{2}Y_{cu}^{\prime }\right)$$

where $Y_{cu}^{\prime }={\bar{P}}_{c}G_{cu}^{c}\left(1-\sigma_{cu}^{2}\right)\eta_{p}/\left(\sigma^{2}+{\bar{P}}_{c}G_{cu}^{c}\sigma_{cu}^{2}\eta_{p}\right)$.

For a system with perfect CSI available, the long-term average rate model can be easily obtained from (23) and (24) by dropping $\eta_{b}$, $\eta_{p}$ and with $\sigma_{cu}^{2}=0$.

The AC optimization problem can be defined as follows. By considering a suitably long time interval, let us denote with $\alpha_{u}$ the fractional amount of RBs per slot allocated to CU *u* and with $\beta_{bv}^{\left(u\right)}$ the fractional amount of RBs per slot that CU *u* shares with SU *v* of cell *b* with b>0. The following resource sharing constraint

(26) $$\sum_{b=1}^{F}\sum_{v=1}^{K_{s}}\beta_{bv}^{\left(u\right)}\leq \alpha_{u},\;\forall u$$

must hold if we assume that one CU can share a RB with no more than one SU. Moreover, the total amount of allocated RBs per slot must not exceed the total number *S*, i.e.,

(27) $$\sum_{u=1}^{K_{c}}\alpha_{u}\leq S$$

The average rate achieved by the CU *u*, when it shares the RBs with a set of SUs having $\beta_{bv}^{\left(u\right)}>0$, must be larger than the rate requirement $q_{0u}$, i.e.,

(28) $$\left(\alpha_{u}-\sum_{b=1}^{F}\sum_{v=1}^{K_{s}}\beta_{bv}^{\left(u\right)}\right)r_{0u}^{\prime }+\sum_{b=1}^{F}\sum_{v=1}^{K_{s}}\beta_{bv}^{\left(u\right)}r_{0u}^{\left(bv\right)}\geq q_{0u}$$

where the first term accounts for the rate achieved in orthogonal mode. On the other hand, the rate achieved by the SU *v* of cell b>0 must be larger than the rate requirement $q_{bv}$, i.e.,

(29) $$\sum_{u=1}^{K_{c}}\beta_{bv}^{\left(u\right)}r_{bv}^{\left(0u\right)}\geq q_{bv}$$

if we assume that the CUs occupy all the RBs and the SUs operate in sharing mode only, as already stated in Section 2.

The CU *u* is an admitted user if and only if $\alpha_{u}>0$. The SU *v* of cell b>0 is an admitted user if and only if $\sum_{u=1}^{K_{c}}\beta_{bv}^{\left(u\right)}>0$. Therefore, we have

(30) $$z_{0u}=I\left(\alpha_{u}>0\right),\;z_{bv}=I\left(\sum_{u=1}^{K_{c}}\beta_{bv}^{\left(u\right)}>0\right)$$

where I(.) is the indicator function. Given the previously presented resource constraints, the AC algorithm looks for the solution of the following mixed integer linear problem:

(31a) $$\begin{array}{cc} \max_{\alpha ,\beta }\; & U(z)\;\;\; \end{array}$$

(31b) $$\begin{array}{cc} s.t.\; & \;(26),\;(27),\;(28),\;(29),\;(30) \end{array}$$

where $\alpha =\{\alpha_{u}\geq 0,\;u=1,\ldots ,K_{c}\}$ and $\beta =\{\beta_{bv}^{\left(u\right)}\geq 0,\;b=1,\ldots ,F,\;v=1,\ldots ,K_{s},\;u=1,\ldots ,K_{c}\}$. The AC maximizes the total revenue of the service provider, and when the revenue from each user is proportional to the required average rate, it maximizes the network capacity.

It is important to note that the main outcome of the AC algorithm is the set z of admission variables, which is the input of RA. The sets α and β just define the optimal average amount of allocated resources predicted by the AC algorithm. These sets are not considered by the RA algorithm, since it uses the estimated short-term channel gains to optimize the resource allocation.

Given the results of the AC algorithm, z, we use the admission rate as a metric to characterize AC performance, defined as

(32) $$AR_{c}=\sum_{u=1}^{K_{c}}z_{0u}/K_{c}$$

(33) $$AR_{s}=\sum_{c=1}^{F}\sum_{u=1}^{K_{s}}z_{cu}/\left(FK_{s}\right)$$

for CUs and SUs, respectively. Another relevant metric is the network capacity expected after AC, given by

(34) $$C_{E}=\sum_{c=1}^{F}\sum_{u=1}^{K_{s}}z_{cu}q_{cu}+\sum_{u=1}^{K_{c}}z_{0u}q_{0u}$$

which is evaluated in [33].

## 7. Simulation Results

In this section, we present the numerical results obtained through simulations using MATLAB^®^ code to implement system models and algorithms. We investigate the effects of pilot-symbol-assisted channel estimation, which provides imperfect CSI, on the framework of a QoS-aware RA jointly working with admission control. In particular, we compare the results, in terms of network capacity and ability to fulfill single-user QoS requirements, obtained from (i) RA and AC algorithms that are unaware of channel estimation mechanisms (CE-unaware strategies), and (ii) RA and AC algorithms proposed in this paper that take into account channel estimation error and overhead (CE-aware strategies). The penalty with respect to the perfect CSI case is also evaluated. The CE-unaware strategies run the RA and AC algorithms designed for the perfect CSI case to select admitted users and to perform subchannel and power allocation. However, the actual performance of the system is evaluated by considering pilot-symbol overhead and channel estimation error. It is also noted that the CE-unaware RA algorithm is the algorithm proposed in [18] for a single-cell network with perfect CSI knowledge and suitably extended for use in our multi-cell heterogeneous network. We expect that imperfect CSI, beyond performance degradation, makes the control of interference and the provision of a fair QoS to all users difficult. The proposed CE-aware RA and AC algorithms should cope with this challenge.

The simulations are carried out considering the downlink of a two-tier HetNet with system parameters defined as in Table 2. To set up the parameters, 4G numerology is considered, as in [33]. The length of the RA frame is arbitrarily chosen to three slots. For pilot-aided channel estimation, four pilot symbols per RB are used. As the RB has 12×7 symbols, we set μ=1/21. Parameters $A_{1},A_{3}$ for the link model are set as in [28]. The utility weights are taken as uniformly distributed in [0.5, 1] for CUs and in [0, 0.5] for SUs in order to prioritize CUs over SUs.In all simulations, the multipath propagation in the wireless channel is modeled with an exponentially decaying PDP, as in [29], with a decay time of 1 μs. In the results, we use different values for the number *F* of small cells in the HetNet and for the two high-sensitivity parameters of the system, i.e., the number of users $K_{s}$ per cell, and the amplification coefficient α of pilot symbols, which is directly related to the power efficiency of the channel estimation process.

### 7.1. Admission Rate

**Figure 4:**
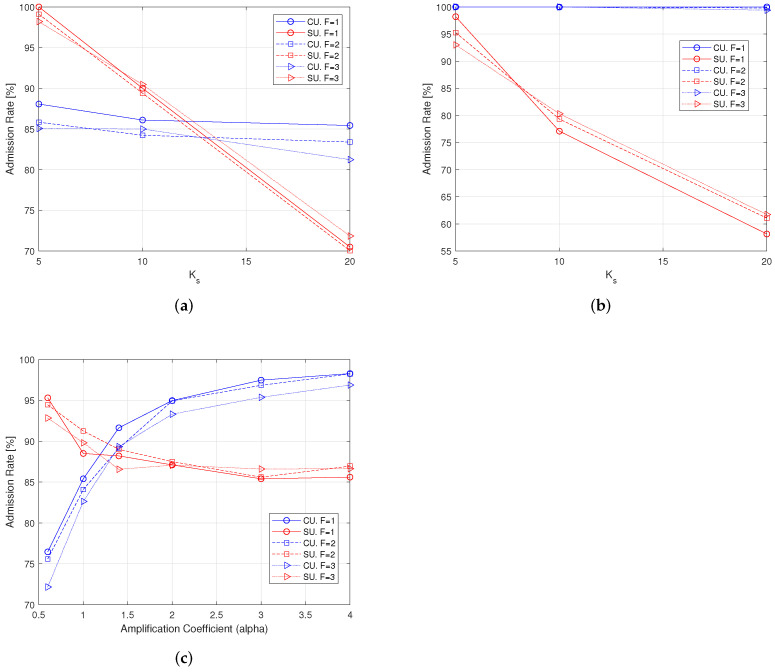
Admission rate with $K_{c}$ = 30 users, different values of *F* and $K_{s}$, 100 iterations. (**a**) Varying $K_{s}$, α = 1, CE-aware; (**b**) varying $K_{s}$, α = 1, CE-unaware; (**c**) varying α, $K_{s}$ = 10, CE-aware.

We first investigate the behavior of AC by looking at the admission rate for both CUs and SUs, which is shown in the plots of Figure 4. The AC outcomes determine the operation setup (load) of the network, which also influences the behavior of the RA.

We observe from Figure 4a,b that by increasing the number of SUs, the admission rate decreases as expected. The CUs, which are considered as primary users in the system with large weights (service revenue) in the utility function, have admission rates approaching 90–100%. However, the CE-aware scheme, which takes channel estimation error into account, reduces the admission rate of CUs and admits more SUs that usually experience a better signal-to noise ratio, due to the small link distance. Admission rate variations are around 10–15%, When the power allocated to the pilots increases (see Figure 4c), the channel estimation error decreases, and the CE-aware scheme admits more CUs with a slight decrease of SUs. This is due to the fact that the utility weights of CUs are larger than those of SUs: the revenue is therefore maximized by admitting more CUs than SUs when the link SINR increases.

In the next subsection, we discuss the effects of RA algorithms on the QoS performance of admitted users.

### 7.2. Average Rate

**Figure 5:**
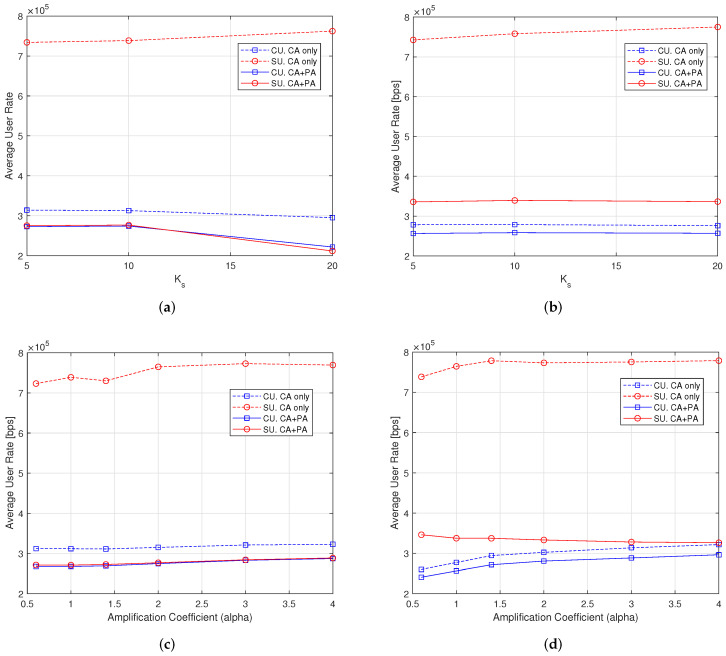
Average user rate with *F* = 1, $K_{c}$ = 30 users, 100 iterations. (**a**) Varying $K_{s}$, α = 1, CE-aware; (**b**) varying $K_{s}$, α = 1, CE-unaware; (**c**) varying α, $K_{s}$ = 10, CE-aware; (**d**) varying α, $K_{s}$ = 10, CE-unaware.

In Figure 5, the average user rate provided to both CUs and SUs after subchannel allocation (CA) only and after CA plus power allocation (PA) is investigated. We observe that, in general, the suboptimal CA algorithm alone is not able to equalize the rates provided to the single users. SUs have better channel and interference conditions and achieve larger unequal rates. Note that the average rates are larger when rate distribution is more unfair. However, after the PA process, the average rates of CUs and SUs become similar and even equal when CE-aware strategies are used. It is interesting to note that the QoS requirements of the users (256 kbps, as in Table 2) are almost satisfied.

From Figure 5a,b, we note that the average rate is almost independent of the number of candidate SUs, which means that the RA algorithm is able to provide the required QoS to the set of users carefully admitted by the AC algorithm. Moreover, we note that the CE-aware algorithm is able to equalize the rates of CUs and SUs, whereas the CE-unaware algorithm still works, but the SU rate is 20% larger than the CU rate. From Figure 5c,d, we note that the achieved average rate increases when the power of the pilot symbols increases for both CUs and SUs with the CE-aware algorithms. In this case, the positive effect of channel estimation error reduction overcomes the power efficiency penalty. With the CE-unaware algorithms, the SU rate decreases when the CU rate increases, but more power on the pilots makes a noticeable contribution to equalizing the rates.

### 7.3. Rate Dispersion Index

The Rate Dispersion Index (RDI) indicates if the set of user rates is clustered or dispersed. It is defined as the difference between maximum and minimum values in the set, normalized to the average value of the set.

**Figure 6:**
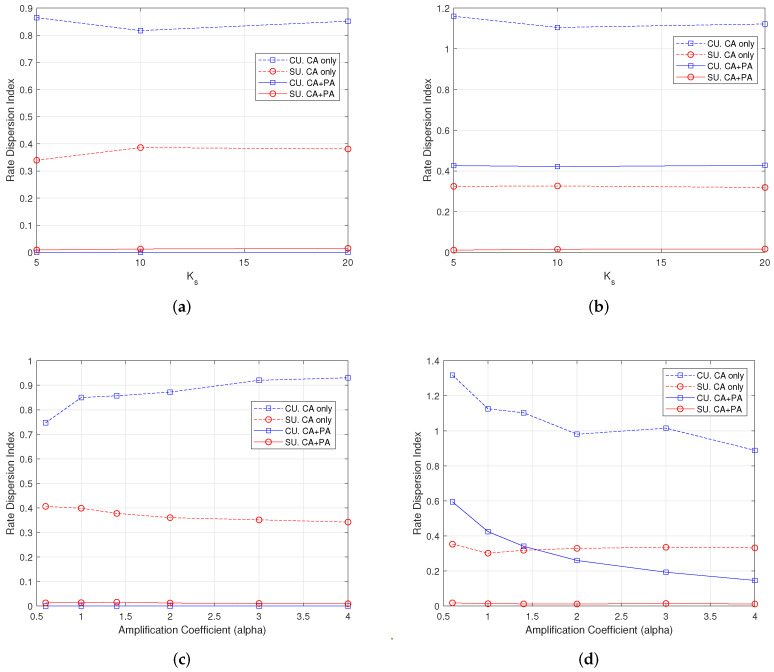
Rate dispersion index with *F* = 1, $K_{c}$ = 30 users, 100 iterations. (**a**) Varying $K_{s}$, α = 1, CE-aware; (**b**) varying $K_{s}$, α = 1, CE-unaware; (**c**) varying α, $K_{s}$ = 10, CE-aware; (**d**) varying α, $K_{s}$ = 10, CE-unaware.

In Figure 6, the plots show the RDI for the same cases of Figure 5. The values of this index clearly confirm the findings and the comments in the previous subsection. We can see in Figure 6a,c that the index goes near to zero for both CUs and SUs when the CE-aware RA algorithm with both CA and PA is used. When RDI = 0, the same average rate (perfect fairness) is provided to all users. The index raises to 0.4 in Figure 6b for CUs when the best CE-unaware RA scheme is used, whereas the RA scheme still works well for the SUs. This is due to the major load of the macrocell with respect to the femtocells. The RDI value for the CUs is found to be quite sensitive to the power allocated to pilots, as shown in Figure 6d, and goes below 0.2 when α>3.

### 7.4. Outage Rate

**Figure 7:**
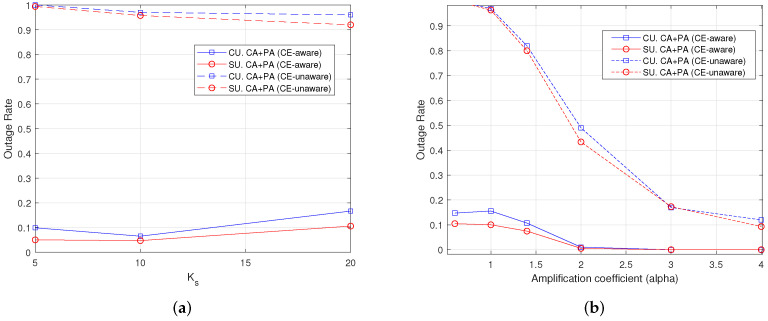
Outage rate with *F* = 1, $K_{c}$ = 30 users, 100 iterations. (**a**) Varying $K_{s}$, α = 1; (**b**) varying α, $K_{s}$ = 10.

The outage rate is the simulative evaluation of the probability that the rate provided to any user is less than the requirement, i.e., $R_{cu}<q_{cu}$, ∀c,u, which is an important metric. In addition, the results for this metric confirm the previous findings and the good behavior of CE-aware algorithms with respect to CE-unaware algorithms. We can see in Figure 7a,b that the outage rate is below 0.2 and quite balanced among CUs and SUs—slightly larger for CUs. It goes to 0 when the power of pilot symbols increases. The outage rate for CUs raises to nearly 1 in Figure 7a when a CE-unaware RA scheme is used. It is also quite sensitive to the power allocated to pilots, as shown in Figure 7b, and goes below 0.2 when α>3, approaching 0 in the CE-aware scheme.

### 7.5. Average Sum-Rate

**Figure 8:**
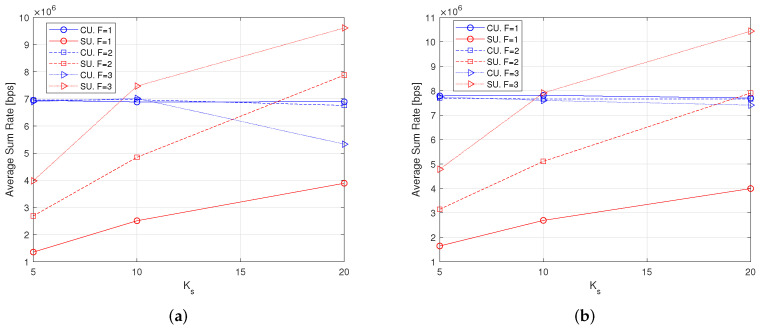

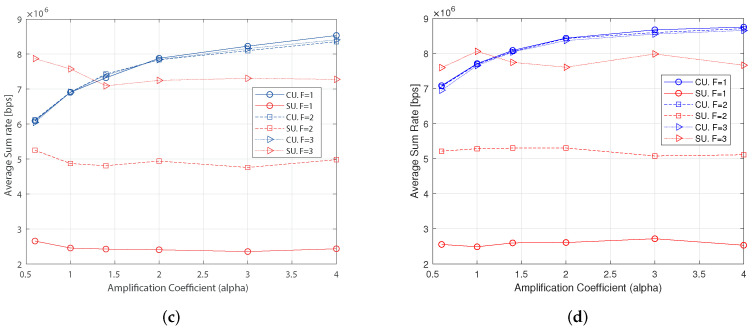
Average Sum-Rate with $K_{c}$ = 30 users, 100 iterations. (**a**) Varying $K_{s}$, α = 1, CE-aware; (**b**) Varying $K_{s}$, α = 1, CE-unaware; (**c**) Varying α, $K_{s}$ = 10, CE-aware; (**d**) Varying α, $K_{s}$ = 10, CE-unaware.

The average sum-rate provides information on the network capacity. Figure 8 shows the average sum-rate for both macrocells and small-cells separately, for different values of *F* from 1 to 3. The RA algorithms include CA and PA. When the number $K_{s}$ of SUs increases, the average sum-rate also increases, because the number of served SUs increases (even if the admission rate decreases, as shown in Figure 4). However, the average sum-rate of the macrocell is preserved (with the exception of the case with F=3 and $K_{s}=20$). When the power of pilot symbols increases, with α up to 4, the sum-rate of the macrocell increases, which is in part due to the increase of the admission rate (see Figure 4). We can also note from the plots in Figure 8 that the sum-rate is slightly larger for the CE-unaware scheme, but this is obtained by sacrificing user rate fairness, as shown by the previous results.

### 7.6. Comparison with Perfect CSI

**Figure 9:**
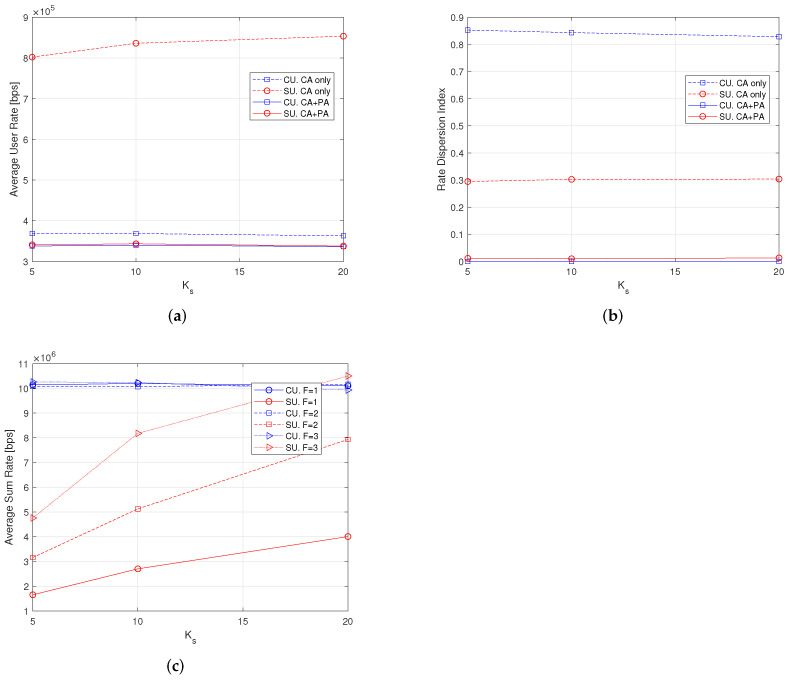
Average user rate, rate dispersion index and average sum-rate with Perfect CSI with $K_{c}$ = 30 users, 100 iterations. (**a**) Varying $K_{s}$, *F* = 1; (**b**) Varying $K_{s}$, *F* = 1; (**c**) Varying $K_{s}$ and *F*.

We finally compare the performance of the systems with pilot symbol-assisted channel estimation and with perfect CSI. This allows us to evaluate the penalty due imperfect CSI in HetNets when AC and RA algorithm are CE-aware. The plots in Figure 9 show the average user rate, the RDI and the average sum-rate, when varying the number $K_{s}$ of SUs, for the perfect CSI case. These plots can be compared with the plots in Figure 5a, Figure 6a and Figure 8a. We can see that average user rate penalty is around 20%, whereas the average sum rate penalty is nearly 30% for the macrocell and only a few percent for femtocells. The RDI is always near to zero.

## 8. Conclusions

In this paper, QoS-aware RA jointly working with AC over a two-tier HetNet scenario has been investigated in the presence of pilot-symbol-aided channel estimation, which introduces rate and power penalties, and a channel estimation error. A novel RA algorithm has been designed by including the effects of pilot-aided channel estimation, as a suboptimal solution of a sum-rate maximization problem with proportional rate constraints and per cell power constraints. The results obtained from the proposed algorithms have been compared with the results obtained from algorithms not aware of pilot-aided channel estimation effects and with the scenario with perfect CSI. The simulation results have shown that the proposed RA algorithms (with two iterations in power allocation algorithm) are able to distribute the same proportional rate to all CUs and SUs, with almost zero dispersions, even in the presence of channel estimation error. For the CE-unaware algorithms, it is much more difficult to equalize the average rates provided to all users, especially for the CUs in high load conditions that achieve a RDI above 0.4. The comparison with the performance obtained in the perfect-CSI scenario has shown that the average user rate penalty is around 20%, whereas the average sum-rate penalty is nearly 30% for the macrocell and only a few percent for the small cells.

## Figures and Tables

**Table 1:** Main symbols and acronyms.

Symbols	Description
$r_{c}$	Macro-cell radius
$r_{f}$	Small-cell radius
$K_{c}$	Number of CUs per cell
$K_{s}$	Number of SUs per cell
*F*	Number of small-cells
$G_{cu}^{b}$	Average channel gain between user *u* of cell *c* and BS/AP *b*
$h_{cu,s}^{b}$	Fading gain between user *u* of cell *c* and BS/AP *b* on subchannel *s*
ΔB	Subchannel bandwidth
$w_{cu}$	Utility weights
$q_{cu}$	Required average rate for user *u* of cell *c*
*S*	Subchannels per slot
*T*	Slots per frame
$\gamma_{cus}^{\left(bv\right)}$	SINR for the user *u* of cell *c*, when it shares the channel with user *v* of cell *b* on subchannel *s*
$R_{cu}$	Average rate of user *u* of cell *c* over one slot
$P_{T}^{c}$	Power budget per frame in cell *c*
$P_{us}^{c}$	Power spent by BS/AP *c* for user *u* on subchannel *s*
$a_{cus}$	Allocation variable of user *u* of cell *c* for subchannel *s*
$\sigma^{2}$	Noise power per subchannel
$\sigma_{cu}^{2}$	Power of estimation error for user *u* of cell *c*
μ	Number of pilots/Number of data symbols Ratio
**Acronyms**	**Full Name**
RA	Resource allocation
AC	Admission control
BS	Base station
AP	Small-cell access point
CU	Cellular user
SU	Small-cell user
CSI	Channel state information
PDP	Power delay profile
SINR	Signal-to-interference-plus-noise ratio
RB	Resource block

**Table 2:** Simulation parameters.

Variables	Values
Macro-cell radius ($r_{c}$)	500 m
Small-cell radius ($r_{f}$)	20 m
$r_{i}$	150 m
$A_{1}$	2.061
$A_{3}$	0.945
ς	0.4
Subchannel bandwidth (ΔB)	180 KHz
Subchannels per slot (S)	15
Length of RA frame (T)	3
BS/AP power budget ($P_{T}^{c}/T$)	21.7 dBm
Noise power $\left(\sigma^{2}\right)$	$3.360\times 10^{-15}$
Fraction of pilots $\left(\mu =N_{P}/N\right)$	1/21
Minimum av. rate $\left(q_{cu}\right)$	256 kbps
CU utility weights $\left(w_{cu},c=0\right)$	uniform in [0.5, 1]
SU utility weights $\left(w_{cu},c\neq 0\right)$	uniform in [0, 0.5]

## Data Availability

Not applicable.

## Appendix A

The aim of this appendix is to address the derivation of the solution of the power allocation problem in (14). We can start by writing the Lagrangian of the problem as follows:

(A1) $$\begin{array}{c} L\left(P\right)=\sum_{c}\sum_{u}\sum_{s}\eta_{cus}^{*}\log_{2}\left(1+\frac{\Gamma_{cus}}{A_{1}}\right)+\sum_{c}\lambda_{c}\left(\sum_{s}\sum_{u}a_{cus}^{*}P_{us}^{c}-P_{T}^{c}\right)+\\ \sum_{c}\sum_{u>1}\mu_{cu}\left[\sum_{s}\frac{\eta_{c1s}^{*}}{\phi_{c1}}\log_{2}\left(1+\frac{\Gamma_{c1s}}{A_{1}}\right)-\right.\left.\sum_{s}\frac{\eta_{cus}^{*}}{\phi_{cu}}\log_{2}\left(1+\frac{\Gamma_{cus}}{A_{1}}\right)\right]+\\ \sum_{c>0}\mu_{c1}\left[\sum_{s}\frac{\eta_{01s}^{*}}{\phi_{01}}\log_{2}\left(1+\frac{\Gamma_{01s}}{A_{1}}\right)-\right.\left.\sum_{s}\frac{\eta_{c1s}^{*}}{\phi_{c1}}\log_{2}\left(1+\frac{\Gamma_{c1s}}{A_{1}}\right)\right] \end{array}$$

where $\lambda_{c}$, $\mu_{cu}$, with c=0,…,F, $u=1,\ldots ,K_{c}\left(K_{s}\right)$ are the Lagrangian multipliers.

Now, we differentiate with respect to $P_{us}^{c}$ in order to find the conditions of the power variables to achieve the maximum of the Lagrangian. We surely have $P_{us}^{c}=0$ if $a_{cus}^{*}=0$. We can derive $P_{us}^{c}$, when $a_{cus}^{*}=1$, from the set of equations

(A2) $$\begin{array}{c} \frac{\partial L}{\partial P_{1s}^{0}}=\eta_{01s}\log_{2}e\frac{1}{1+\frac{\Gamma_{01s}}{A_{1}}}\frac{1}{A_{1}}\frac{X_{01s}Z_{01s}}{{\left(P_{1s}^{0}Y_{01}+Z_{01s}\right)}^{2}}+\lambda_{0}a_{01s}^{*}\\ \;+\sum_{c>0}\mu_{c1}\frac{\eta_{01s}^{*}}{\phi_{01}}\log_{2}e\frac{1}{1+\frac{\Gamma_{01s}}{A_{1}}}\frac{1}{A_{1}}\frac{X_{01s}Z_{01s}}{{\left(P_{1s}^{0}Y_{01}+Z_{01s}\right)}^{2}}\\ \;+\sum_{u>1}\mu_{0u}\frac{\eta_{01s}^{*}}{\phi_{01}}\log_{2}e\frac{1}{1+\frac{\Gamma_{01s}}{A_{1}}}\frac{1}{A_{1}}\frac{X_{01s}Z_{01s}}{{\left(P_{1s}^{0}Y_{01}+Z_{01s}\right)}^{2}}=0 \end{array}$$

(A3) $$\begin{array}{c} \frac{\partial L}{\partial P_{1s}^{c}}=\eta_{c1s}\log_{2}e\frac{1}{1+\frac{\Gamma_{c1s}}{A_{1}}}\frac{1}{A_{1}}\frac{X_{c1s}Z_{01s}}{{\left(P_{1s}^{c}Y_{c1}+Z_{c1s}\right)}^{2}}+\lambda_{0}a_{c1s}^{*}\\ \;-\mu_{c1}\frac{\eta_{c1s}^{*}}{\phi_{c1}}\log_{2}e\frac{1}{1+\frac{\Gamma_{c1s}}{A_{1}}}\frac{1}{A_{1}}\frac{X_{c1s}Z_{c1s}}{{\left(P_{1s}^{c}Y_{c1}+Z_{c1s}\right)}^{2}}\\ \;+\sum_{u>1}\mu_{cu}\frac{\eta_{c1s}^{*}}{\phi_{c1}}\log_{2}e\frac{1}{1+\frac{\Gamma_{c1s}}{A_{1}}}\frac{1}{A_{1}}\frac{X_{c1s}Z_{c1s}}{{\left(P_{1s}^{c}Y_{c1}+Z_{c1s}\right)}^{2}}=0\\ \;\forall c>0 \end{array}$$

(A4) $$\begin{array}{c} \frac{\partial L}{\partial P_{us}^{c}}=\eta_{cus}^{*}\log_{2}e\frac{1}{1+\frac{\Gamma_{cus}}{A_{1}}}\frac{1}{A_{1}}\frac{X_{cus}Z_{cus}}{{\left(P_{us}^{c}Y_{cu}+Z_{cus}\right)}^{2}}+\lambda_{c}a_{cus}^{*}\\ \;-\mu_{cu}\frac{\eta_{cus}^{*}}{\phi_{cu}}\log_{2}e\frac{1}{1+\frac{\Gamma_{cus}}{A_{1}}}\frac{1}{A_{1}}\frac{X_{cus}Z_{cus}}{{\left(P_{us}^{c}Y_{cu}+Z_{cus}\right)}^{2}}=0\\ \;\forall c,\forall u>1 \end{array}$$

Note that, from Equation (17), in the text,

$$\begin{array}{c} \frac{1}{A_{1}+\Gamma_{cus}}\frac{X_{cus}Z_{cus}}{{\left(P_{us}^{c}Y_{cu}+Z_{cus}\right)}^{2}}=\frac{1}{A_{1}+\frac{P_{us}^{c}X_{cus}}{\left(P_{us}^{c}Y_{cu}+Z_{cus}\right)}}\frac{X_{cus}Z_{bvs}}{{\left(P_{us}^{c}Y_{cu}+Z_{bvs}\right)}^{2}}=\\ \frac{X_{cus}Z_{bvs}}{A_{1}{\left(P_{us}^{c}Y_{cu}+Z_{cus}\right)}^{2}+P_{us}^{c}X_{cus}\left(P_{us}^{c}Y_{cu}+Z_{cus}\right)}=\frac{X_{cus}Z_{cus}}{{\left(P_{us}^{c}\right)}^{2}\alpha_{cus}+P_{us}^{c}\beta_{cus}+A_{1}{\left(Z_{cus}\right)}^{2}} \end{array}$$

where:

$$\begin{array}{c} \alpha_{cus}=Y_{cu}\left(A_{1}Y_{cu}+X_{cus}\right),\\ \beta_{cus}=Z_{cus}\left(X_{cus}+2A_{1}Y_{cu}\right), \end{array}$$

Therefore, all the Equations (A2)–(A4) can be manipulated to assume the form

(A5) $$\eta_{cus}^{*}\gamma_{cu}\frac{X_{cus}Z_{cus}\log_{2}e}{{\left(P_{us}^{c}\right)}^{2}\alpha_{cus}+P_{us}^{c}\beta_{cus}+A_{1}{\left(Z_{cus}\right)}^{2}}=-\lambda_{c}a_{cus}^{*}$$

where $\gamma_{cu}$ is a constant parameter depending on $\phi_{cu}$ and the set of Lagrangian multipliers $\mu_{cu}$ only. A first important conclusion can be derived from (A5): if the user *u* of the cell *c* has two or more allocated subcarriers, the following relationship applies for any pair s,r of allocated subcarriers, i.e., with $a_{cus}^{*}=a_{cur}^{*}=1$:

$$\begin{array}{c} \frac{X_{cus}Z_{cus}}{{\left(P_{us}^{c}\right)}^{2}\alpha_{cus}+P_{us}^{c}\beta_{cus}+A_{1}{\left(Z_{cus}\right)}^{2}}=\frac{X_{cur}Z_{cur}}{{\left(P_{ur}^{c}\right)}^{2}\alpha_{cur}+P_{ur}^{c}\beta_{cur}+A_{1}{\left(Z_{cur}\right)}^{2}} \end{array}$$

which is the Equation (18) in the text. Similarly to [18], this is the “waterfilling” equation for the power allocated to each user across subcarriers, when channel estimation error is taken into account for resource allocation.

If the power allocated over one subcarrier, e.g., subcarrier *r*, is known, we can immediately find the power allocated to any other subcarrier *s* of the same user by solving the following equation with respect to $P_{us}^{c}$:

$$\begin{array}{c} {\left(P_{us}^{c}\right)}^{2}\alpha_{cus}+P_{us}^{c}\beta_{cus}+A_{1}{\left(Z_{cus}\right)}^{2}=\frac{X_{cus}Z_{cus}}{X_{cur}Z_{cur}}\left[{\left(P_{ur}^{c}\right)}^{2}\alpha_{cur}+P_{ur}^{c}\beta_{cur}+A_{1}{\left(Z_{cur}\right)}^{2}\right] \end{array}$$

This is a quadratic equation whose solution is given by $P_{us}^{c}=\frac{-b\pm \sqrt{b^{2}-4ac}}{2a}$, where

$$\begin{array}{c} a=\alpha_{cus},\\ b=\beta_{cus},\\ c=A_{1}Z_{cus}^{2}-\frac{X_{cus}Z_{cus}}{X_{cur}Z_{cur}}\left[{\left(P_{ur}^{c}\right)}^{2}\alpha_{cur}+P_{ur}^{c}\beta_{cur}+A_{1}Z_{cur}^{2}\right] \end{array}$$

By introducing a more general notation with $P_{ur}^{c}=p$, we can express the solution as $P_{us}^{c}=F_{us}^{c}\left[p,r\right]$, where

(A6) $$\begin{array}{c} F_{us}^{c}\left[p,r\right]=\frac{\beta_{cus}}{2\alpha_{cus}}\cdot \max\left(0,\sqrt{1+4\left[\frac{\left(\alpha_{cur}p^{2}+\beta_{cur}p+A_{1}Z_{cur}^{2}\right)\frac{X_{cus}Z_{cus}}{X_{cur}Z_{cur}}-A_{1}Z_{cus}^{2}}{\beta_{cus}^{2}/\alpha_{cus}}\right]}-1\right) \end{array}$$

which also takes the constraint $P_{us}^{c}\geq 0$ into account.
